# Development of Lower Extremity Strength in Ambulatory Children With Bilateral Spastic Cerebral Palsy in Comparison With Typically Developing Controls Using Absolute and Normalized to Body Weight Force Values

**DOI:** 10.3389/fneur.2021.617971

**Published:** 2021-03-19

**Authors:** Nicolaos Darras, Eirini Nikaina, Magda Tziomaki, Georgios Gkrimas, Antigone Papavasiliou, Dimitrios Pasparakis

**Affiliations:** ^1^Gait & Motion Analysis Center, ELEPAP, Athens, Greece; ^2^First Department of Pediatrics, “Aghia Sophia” Children's Hospital, University of Athens, Athens, Greece; ^3^Laboratory of Neuromuscular and Cardiovascular Study of Motion – LANECASM, Athens, Greece; ^4^Department of Pediatric Neurology IASO Children's Hospital, Athens, Greece; ^5^Pediatric Orthopaedic Department, Athens Medical Center, Athens, Greece

**Keywords:** strength, cerebral palsy, lower limb, diplegia, quadriplegia, children and adolescents

## Abstract

This cross-sectional study aimed to examine the development of lower limb voluntary strength in 160 ambulatory patients with bilateral spastic cerebral palsy (CP) (106 diplegics/54 quadriplegics) and 86 typically developing (TD) controls, aged 7–16 years. Handheld dynamometry was used to measure isometric strength of seven muscle groups (hip adductors and abductors, hip extensors and flexors, knee extensors and flexors, and ankle dorsiflexors); absolute force (AF) values in pounds were collected, which were then normalized to body weight (NF). AF values increased with increasing age (*p* < 0.001 for all muscle groups), whereas NF values decreased through adolescence (*p* < 0.001 for all muscle groups except for hip abduction where *p* = 0.022), indicating that increases in weight through adolescence led to decreases in relative force. Both AF and NF values were significantly greater in TD subjects when compared with children with CP in all muscle and all age groups (*p* < 0.001). Diplegics and quadriplegics demonstrated consistently lower force values than TD subjects for all muscle groups, except for the hip extensors where TD children had similar values with diplegics (*p* = 0.726) but higher than quadriplegics (*p* = 0.001). Diplegic patients also exhibited higher values than quadriplegics in all muscles, except for the knee extensors where their difference was only indicative (*p* = 0.056). The conversion of CP subjects' force values as a percentage of the TD subjects' mean value revealed a pattern of significant muscle strength imbalance between the CP antagonist muscles, documented from the following deficit differences for the CP muscle couples: (hip extensors 13%) / (hip flexors 32%), (adductors 27%) / (abductors 52%), and (knee extensors 37%) / (knee flexors 53%). This pattern was evident in all age groups. Similarly, significant force deficiencies were identified in GMFCS III/IV patients when compared with TD children and GMFCS I/II patients. In this study, we demonstrated that children and adolescents with bilateral CP exhibited lower strength values in lower limb muscles when compared with their TD counterparts. This difference was more prevalent in quadriplegic patients and those with a more severe impairment. An important pattern of muscle strength imbalance between the antagonist muscles of the CP subjects was revealed.

## Introduction

Muscle weakness is a major component of cerebral palsy (CP) which contributes to functional disability. Lower limb strength has been correlated with gait deficits and pathological walking patterns (1, 2), while its relationship to joint kinetics has been further described (3, 4). Children with spastic CP (diplegia and hemiplegia) have been found to be weaker than typically developing (TD) children in several studies (3, 5, 6). Muscle weakness is evident even in ambulatory children with mild CP; severe loss of strength has been documented in non-ambulating children with severe CP (7). In a comparison of knee and ankle spasticity and strength in 60 children with spastic diplegia with controls, more distal than proximal involvement in the lower extremities was also demonstrated (6). Stackhouse et al. found that children with spastic diplegic CP (7–13 years) produced 56 and 73% less knee extensor and ankle plantar–flexor force, respectively, compared with participants without disabilities (8). While the aforementioned studies demonstrated weakness in isolated muscle groups, Thompson et al. examined the degree and distribution of weakness in multiple muscles (six muscle groups) in 50 ambulant children with spastic diplegia at Gross Motor Function Classification System (GMFCS) levels I to III and compared them with 15 control children of similar age (9). All muscle groups were significantly weaker in children with CP than in healthy controls (*p* < 0.05) except for the hip extensors. Strength ranged from 43 to 90% of control values depending on the muscle group, with the knee extensors being the relatively weakest group of all. There was significant reduction in strength in all muscle groups with increasing walking difficulty from GMFCS level I to level III. The greatest difference in strength between independent walkers and those dependent on walking aids was in the hip abductors and knee extensors at 30°, which are key muscle groups in sagittal and coronal plane walking stability. In 2014, Davids et al. (10), prospectively examined case series of 255 diplegic children aged 8–19 years. They demonstrated that while the strength of lower extremities increased significantly for the entire group, strength normalized for weight significantly declined with age without important differences among GMFCS levels (10). There was a 90% chance for independent ambulation (GMFCS levels I and II) when strength normalized for weight was 49% predicted relative to TD children.

The ultimate goal of the studies that describe the degree and distribution of weakness in CP was to determine treatment goals and interventions that may result in strength gains and functional improvements; this has proven difficult to accomplish (11). On the contrary, even though about 50–80% of individuals with CP are able to ambulate in some way, there is loss of ambulatory skills in some with age, particularly in those at GMFCS levels III and IV (12, 13); this may be related to the pattern and evolution of muscle weakness they exhibit. Recent evidence as to this matter remains controversial and suggests that muscle strengthening may not result in functional improvements (14). However, in a pragmatic setting, the clinician, the patient, and the family have to explore interventions that are likely to conserve the ability to ambulate and the current level of motor function or alternatively devote time and energy toward directions that may improve participation even with deteriorating motor skills. For this reason, the exploration of muscle strength parameters that evolve with age particularly in different muscle groups remains of interest.

The goals of this study were to compare the pattern of changes in muscle strength between ambulatory patients with bilateral spastic CP and TD subjects between the ages of 7 and 16 years, and also, to examine if specific differences exist in patients with GMFCS levels III/IV as opposed to independent walkers (GMFCS levels I/II). Measuring muscle strength differences in patient groups of different ages and severity of involvement was expected, among other factors, to determine realistic rehabilitation goals and quantitative monitoring of interventions.

## Methods

In order to study the development of force in different ages, the subjects were divided into five age groups from ages 7–16 with a 2-year interval. TD controls were volunteers attending a private school close to our laboratory. They were all informed that their data would be used for research. The measurement protocol was approved by the private school administration, and there was a collaboration with physical education teachers during the measurements.

The patients with CP were selected from patients referred to our center for gait analysis on the basis of their age and their ability to follow (intellectually and behaviorally) the gait analysis protocol. We excluded children who had had orthopedic surgery or botulinum toxin injections in any muscle group, over the preceding year. All CP subjects or their parents signed an informed consent form to allow the use of their data in research. All patients had BSCP and were further categorized into patients with spastic diplegia or patients with spastic quadriparesis or quadriplegia. GMFCS scale categorization of the patients was also applied to identify muscle groups that were significantly weaker in patients who walked with the use of assistive devices (group B: GMFCS levels III and IV) than in patients who were able to walk independently (group A: levels I and II).

**Figure 1:**
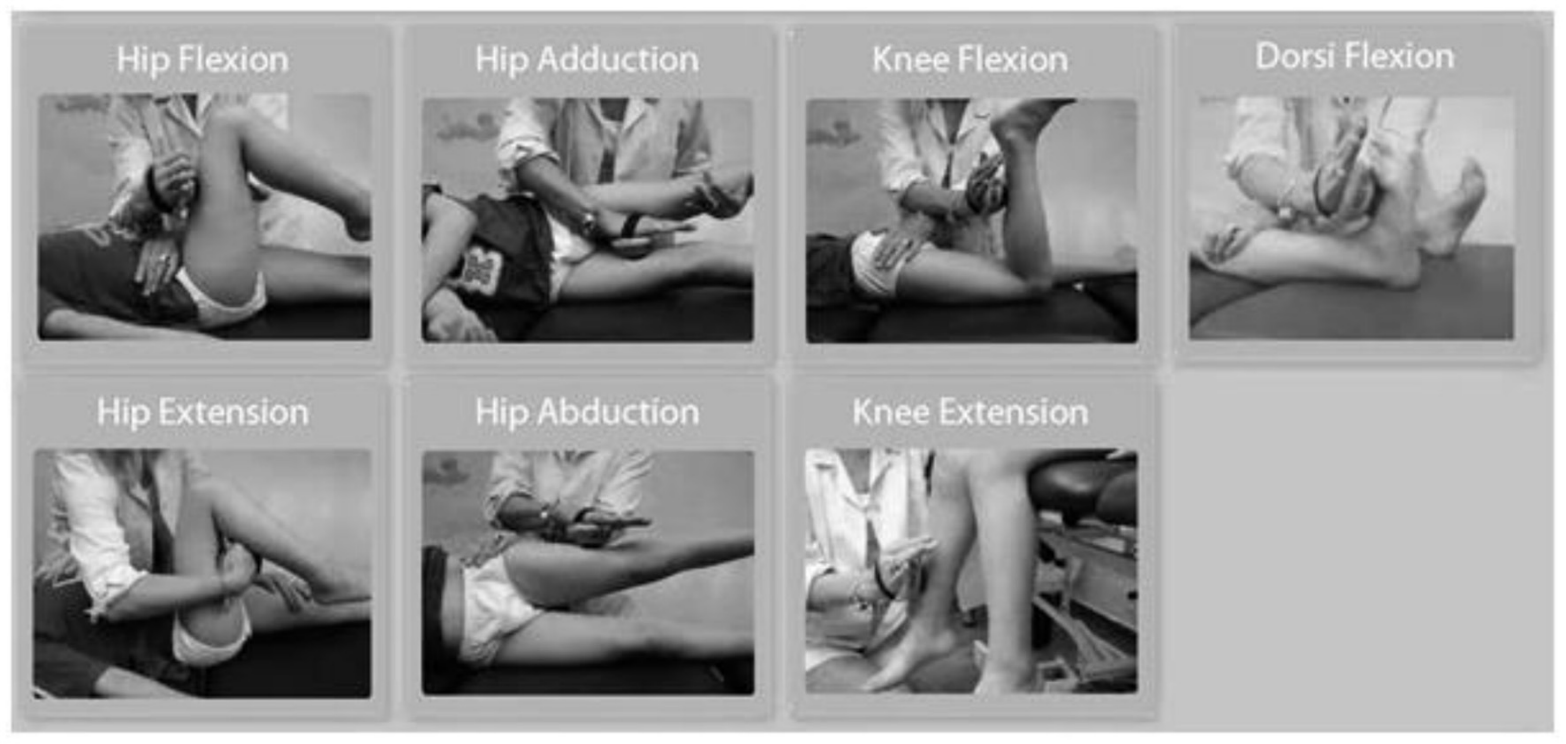
Subject's position and dynamometer application for each muscle group measured.

The force data measurements were collected using a Hoggan microFET2 digital handheld dynamometer. The positioning of the patient and the dynamometer application were standardized as shown in Figure 1. The same examiner performed all measurements in normal subjects and patients. The following seven muscle groups were assessed: hip adductors, hip abductors, hip extensors, hip flexors, knee extensors, knee flexors, and ankle dorsiflexors. Ankle plantar flexors were not included in the study because of the documented difficulties for reliable measurement with the handheld dynamometer (15, 16). All subjects performed three consecutive muscle force measurements with a resting interval of at least 20 s among trials for each muscle group (17, 18). For each trial, the subject was instructed to contract maximally (“as possible”) against the dynamometer for 3 s while encouragement was given by the assessor (measurement type: “make test”), and the maximal dynamometer reading during the 3-s effort was recorded. In cases of subjects where muscle force exceeded the examiner's resistance, an assistant was used to increase the resistance acting over the hand of the examiner. Measurements that could not be performed due to inability of the patients with CP to exhibit resistance were registered and reported as not applicable (N/A) and were inserted as 0 for the analysis. Absolute force (AF) values were registered from the dynamometer and expressed in pounds and then pounds were converted to kilograms (conversion factor 0.45359237). However, absolute strength values may not reflect functional muscle strength due to differences in body weight. Normalized force (NF) values were calculated by dividing AF with body weight and were included in the analysis, to account for these body weight differences. The three measurements for each limb, six measurements in total for each muscle group, were collected and averaged (total measurements = 10332). We chose to analyze the mean value of the three efforts for each muscle group instead of the maximal one (peak value) to take into account the performance variability usually observed in children with CP (18, 19).

CP patients' mean muscle force values were also converted to percentages of the respective TD control mean values. The difference from 100% of the above values was used to define the muscle force deficit that the CP subjects exhibited compared with the TD controls in each muscle group. These deficit values were also used for comparison between the couples of the following antagonist muscle groups: (a) hip extensors/hip flexors, (b) hip adductors/hip abductors, and (c) knee extensors/knee flexors. Equal values indicated a balanced deficit between antagonists, while increased difference indicated disproportional deficit and increased imbalance.

### Statistical Analysis

Demographic data, GMFCS level, and force measurements were available for all study participants. Continuous data were tested for normality using the Kolmogorov–Smirnov test. Normally distributed continuous variables were expressed as mean values (± standard deviation) whereas non-normal data were expressed as median values (range). Categorical data were presented as frequencies and/or percentages. Comparisons across patient groups (typical development, diplegia, quadriplegia) of demographic characteristics, represented as continuous variables, were based on one-way ANOVA if variables were normally distributed (height) and on Kruskal–Wallis test when this was not the case (age, weight). Distribution of gender was recorded for all the participant groups (typical development, diplegia, quadriplegia) and percentages were compared across groups with the chi exact test.

In order to identify factors predicting missing/invalid force measurement values, we performed logistic regression analysis. Factors tested in the univariate analysis were age (categorized in five 2-year groups: 7–8, 9–10, 11–12, 13–14, and 15–16 years), sex (male vs. female), type of the involvement (typical development, diplegia, quadriplegia), and severity of the impairment (GMFCS I–II vs. GMFCS III–IV). Those that were significant or with a *p* ≤ 0.200 were inserted in the multivariate analysis. *p* < 0.05 was set as the significance level.

Comparisons of force values across groups (typical development, diplegia, quadriplegia) were based on one-way ANOVA, except for the ankle dorsiflector force values which were not normally distributed and were compared with the Kruskal–Wallis test. *Post hoc* analyses were performed: Bonferroni correction followed ANOVA; after the Kruskal–Wallis test, we performed pairwise comparisons with the Mann–Whitney *U* test, followed again by Bonferroni correction. Comparisons of force values between the two GMFCS groups (I/II vs. III/IV) were based on *t* tests for all muscles tested, except for ankle dorsiflector force values which were not normally distributed and were compared with the Mann–Whitney *U* test. Stata 13 was used for all analyses.

## Results

### Subject Description

A total of 246 children and adolescents (160 with cerebral palsy and 86 TD subjects) were included in the present study. Participants with cerebral palsy were further categorized into two subgroups: 106 were diplegic (66.25%) and 54 quadriplegic ambulatory (33.75%).

**Figure 2:**
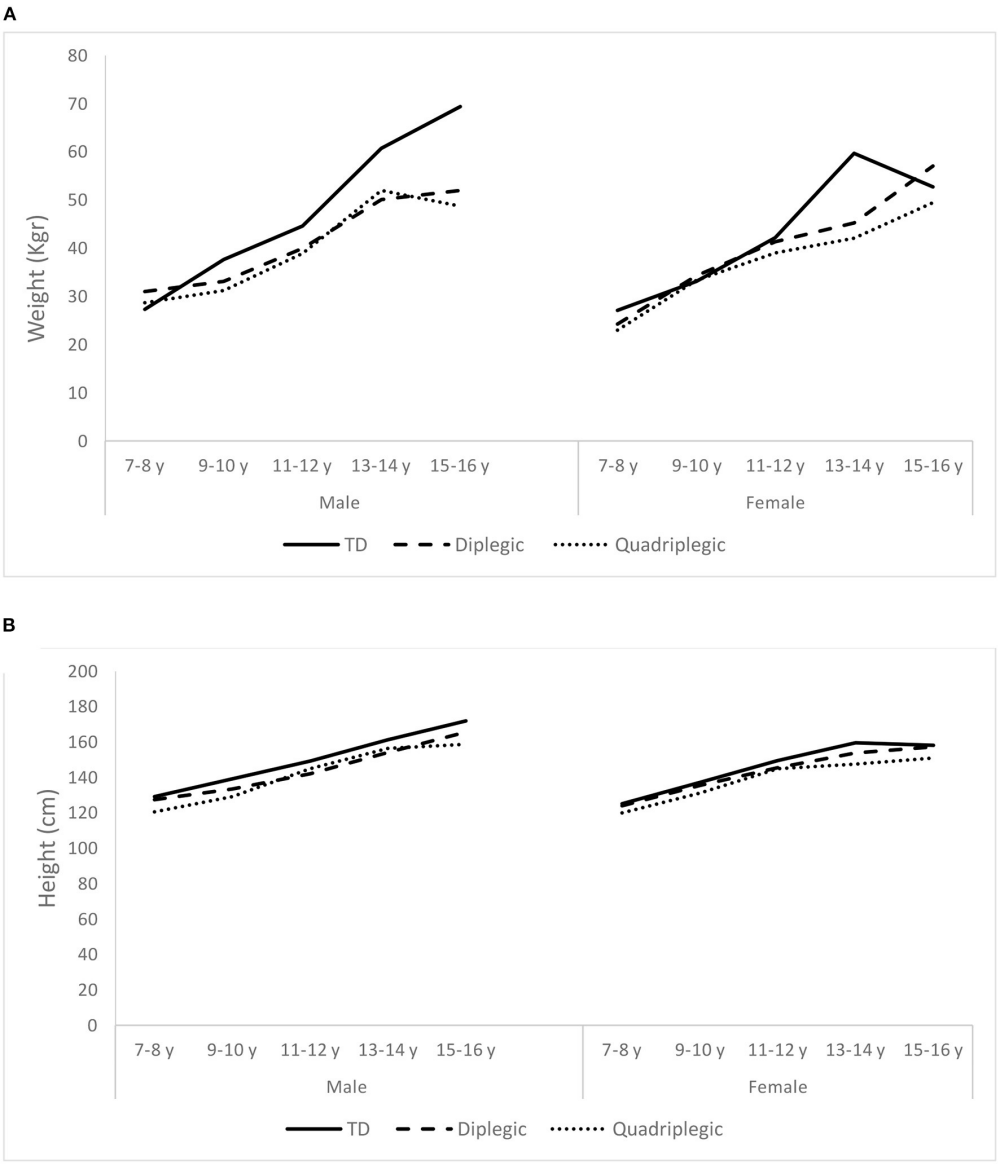
Average weight **(A)** and height **(B)** of the Typically Developing children (TD) and the two patient groups (Diplegic and Quadriplegic), for boys and girls.

Table 1 displays the demographic characteristics of patients and controls. Participants of the two groups were similar in terms of age and sex, but they differed in weight and height. TD participants were heavier than diplegic (*p* = 0.009) and quadriplegic children (*p* = 0.036) and taller than them (*p* = 0.024 and *p* = 0.021, respectively). On the other hand, diplegic, and quadriplegic patients had similar weights and heights (*p* = 0.784 and *p* = 1.000, respectively). When these characteristics were analyzed separately for boys and girls, we discovered that the abovementioned differences were attributed to boys rather than girls. That is, TD and patient girls seemed to have similar weights and heights (*p* = 0.285 and *p* = 0.149, respectively), whereas TD boys were heavier and taller than patient boys (*p* = 0.036 and *p* = 0.032, respectively). Figure 2 displays the changes of weight and height of TD and patients with age.

**Table 1:** Demographic and somatometric characteristics of CP patients and TD children.

	**Diplegia**	**Quadriplegia**	**TD Children**	***p***
	**(*n* = 106)**	**(*n* = 54)**	**(*n* = 86)**	
Age (years)^a^	10 (7–16)	11 (7–16)	11 (7–15)	0.714^*^
Sex (M/F)	58/48	31/23	42/44	0.566
Weight (kg)^a^	35 (20–84)	38.5 (16–59)	42 (22–101)	0.019^*^
Height (cm)^b^	141 ± 15	140 ± 16	147 ± 15	0.008^**^

a *Median values (range)*.

b *Mean values (SD)*.

* *Comparison across groups with Kruskal–Wallis test*.

** *Comparison across groups with one-way ANOVA*.

The distribution of patients in the GMFCS levels were as follows: six patients, all diplegics in GMFCS level I; 115 in level II (86 diplegics/29 quadriplegics); 17 in level III (7 diplegics/10 quadriplegics); and 22 in level IV (7 diplegics/15 quadriplegics).

### Measurement Success

**Figure 3:**
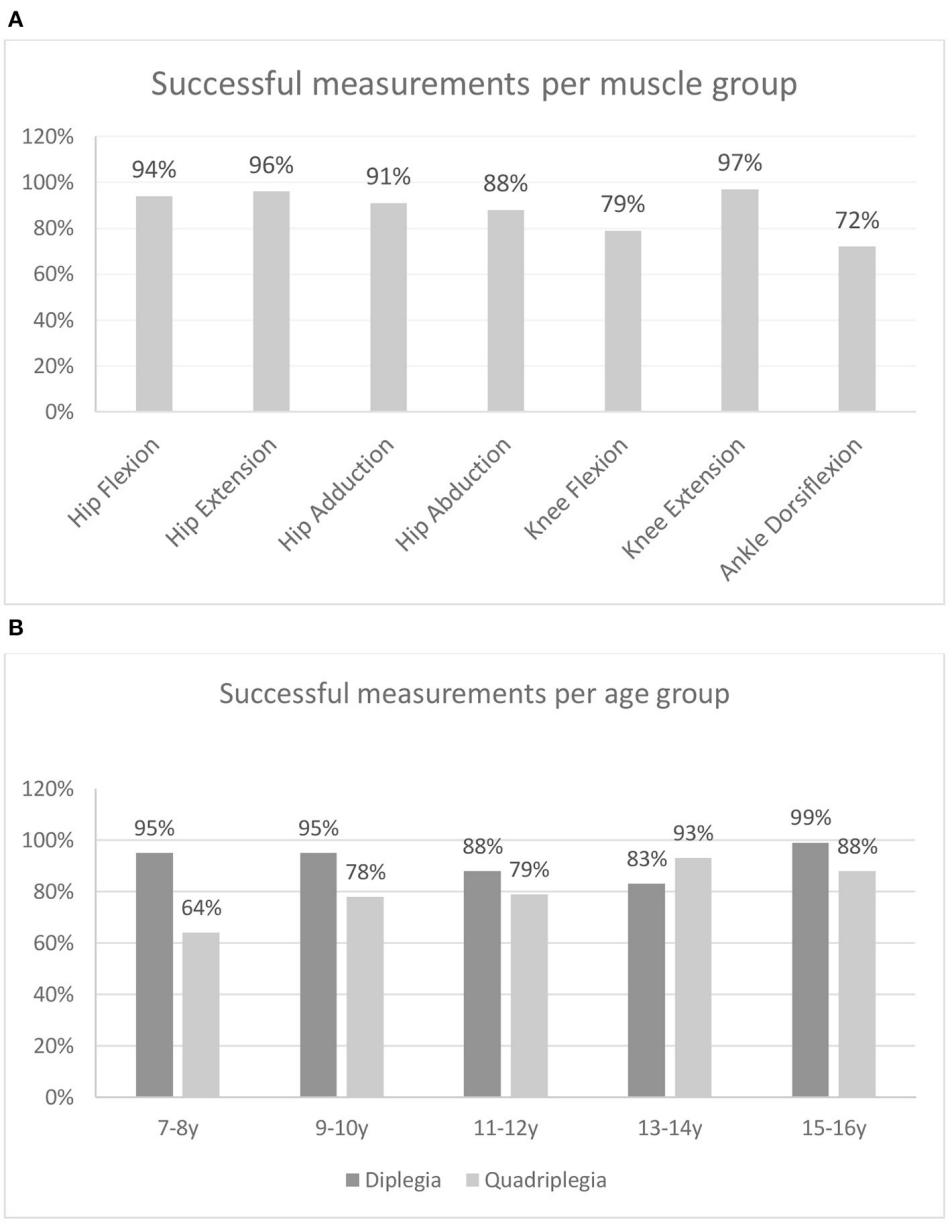
Percentage of successful measurements **(A)** per muscle group and **(B)** per age group for patients with bilateral spastic CP.

In total, 3,444 muscles were finally tested: 2,240 of these were in CP patients. In 256 muscles, measurements were marked as N/A since no attempt of the three performed measurements were valid: in 120 cases, the problem was bilateral; in 10 cases, only the right limb was involved; and in six cases, only the left limb was involved. Only subjects with CP showed N/A values in various muscle groups (*n* = 59). Therefore, 7.4% of the force measurements of the patients were invalid due to the inability of the subjects to perform the tests. In the seven muscle groups tested in the patients with CP, a successful measurement rate of 72–97% was found (Figure 3A). Knee flexion and ankle dorsiflexion showed lower successful measurement rates of 79 and 72%, respectively. Concerning movement that caused difficulty, in 52 cases, it involved the hip (flexion: 10, extension: 7, adduction: 15, abduction: 20); in 39 cases, it involved the knee (flexion: 34, extension: 5); and in 45 cases, the ankle (dorsiflexion). In three patients, one attempt for knee extension (*n* = 1) and hip abduction (*n* = 2) measurement was not valid. However, the other two attempts produced force measurements and the whole measurement was considered valid.

From the successful measurement rates of the two CP groups, across age groups (Figure 3B), it can be observed that for all age groups, except the one of 13–14 years, diplegic subjects showed higher successful measurement rates than quadriplegics. Also, quadriplegic subjects showed substantially higher rates of N/A in younger rather than older age groups. The univariate logistic regression analysis performed to identify patients' characteristics that may be associated with a higher rate of N/A measurements demonstrated that patients with N/A measurements were similar to those with no such results in terms of age (*p* = 0.5382) and sex (*p* = 0.982). On the other hand, quadriplegic patients and those with a more serious involvement (GMFCS III–IV) were more likely to have N/A measurements when compared with diplegics (*p* < 0.001) and those patients with GMFCS I–II (*p* < 0.001). After multivariate logistic regression analysis, quadriplegic children and those with more serious involvement still had more invalid measurements (*p* = 0.002 and *p* = 0.022, respectively).

### Force Results

**Figure 4:**
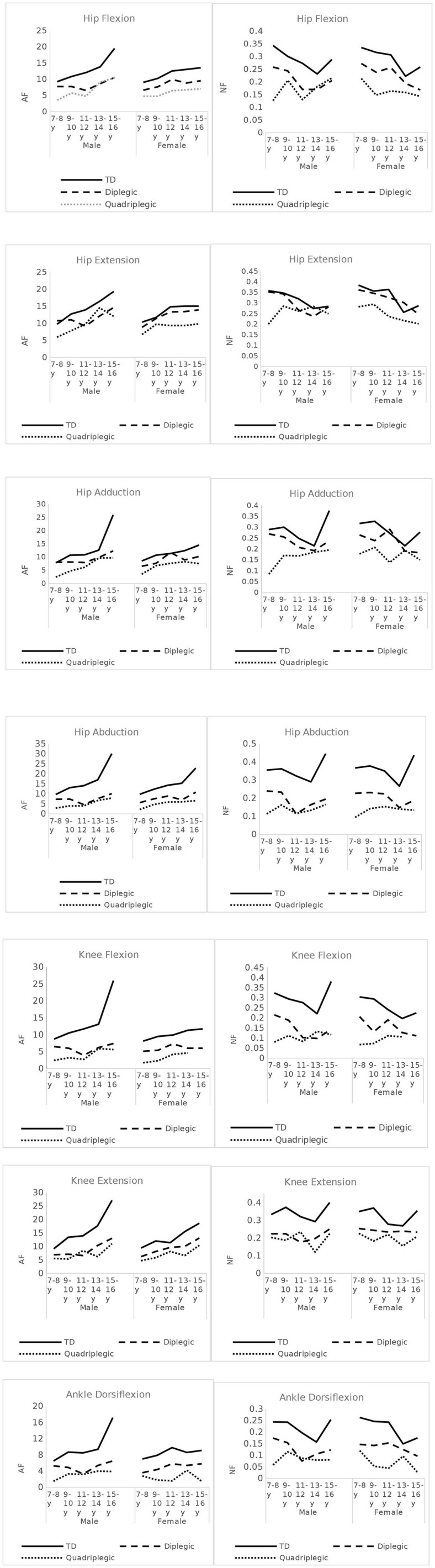
Absolute Force (AF) and Normalized Force (NF) - Force per Kg- values across age-groups, for all muscles tested, in CP patients and TD children.

As demonstrated in the AF graphs (Figure 4), AF values showed a positive (upward) slope, with increasing age. TD children's AF graphs showed a progressive increase in all muscle groups. For TD boys, this increase presented a steep slope especially after 13–14 years. In TD girls, this steep increase was still present, but it was demonstrated earlier (9–10 or 11–12 years of age) for hip flexors, hip extensors, knee flexion, and knee extension and at same age as boys for hip adductors and abductors. For ankle dorsiflexion, we could not display such a pattern. CP patients, of both sexes, had distinguishably lower AF values for most muscle groups. A lower difference between diplegic patients and TD children was presented in hip extension (for girls) and hip adduction (for boys and girls), especially during childhood and to a lesser extent into adolescence. CP children, especially diplegic patients, also demonstrated some steep increases, though with greater variations.

On the contrary, NF values showed a negative (downward) slope. TD children demonstrated a notable decrease at the 13–14 year age group in all measured muscles and for both boys and girls, followed by a sudden increase thereafter (Figure 4). In CP patients, this pattern was not so evident. For diplegic patients, the secondary increase was observed mostly in males. Quadriplegic patients had more varying patterns. As demonstrated in the graphs, bigger differences between TD and diplegic–quadriplegic patients were observed for hip abduction, knee flexion and extension, and ankle dorsiflexion and hip flexion to a lesser degree. For hip extension and hip adduction, NF values especially between TD and diplegic patients were not distinguishable.

Table 2 displays mean force measurements for CP patients and TD children for all muscle groups tested. Hip flexor values were found significantly lower in both diplegic and quadriplegic patients than in TD subjects (*p* < 0.001 for both comparisons), and quadriplegic patients also had lower values than diplegic patients (*p* < 0.001). Furthermore, hip extensor values were also different in the compared groups. In this muscle group, however, TD subjects had similar values with diplegic patients (*p* = 0.726) but higher values than quadriplegics (*p* = 0.001). For this muscle group, force values were also different between quadriplegic and diplegic patients (*p* = 0.001). In hip adduction, the pattern followed that of hip flexion with all participant groups differing from each other (diplegic–TD: *p* = 0.002, quadriplegic–TD: *p* < 0.001, diplegic–quadriplegic: *p* < 0.001). For hip abduction and knee flexion, that was also the case (*p* < 0.001, for all group comparisons). Concerning knee extension, both patient groups differed from TD subjects (*p* < 0.001 for both comparisons), but the difference between quadriplegic and diplegic patients was indicative (*p* = 0.056). Similarly, for ankle dorsiflexion, all groups differed from each other (*p* < 0.001 for all comparisons).

**Table 2:** Comparison of normalized to weight force measurements in CP patients and TD children.

	**Diplegic patients**	**Quadriplegic patients**	**TD children**	***p***
	***(n = 106)***	***(n = 54)***	***(n = 86)***	
Hip flexion^a^	0.222 (±0.085)	0.168 (±0.083)	0.285 (±0.072)	<0.001^*^
Hip extension^a^	0.309 (±0.094)	0.254 (±0.105)	0.324 (±0.073)	<0.001^*^
Hip adduction^a^	0.235 (±0.091)	0.171 (±0.091)	0.279 (±0.073)	<0.001^*^
Hip abduction^a^	0.202 (±0.086)	0.132 (±0.084)	0.348 (±0.091)	<0.001^*^
Knee flexion^a^	0.160 (±0.092)	0.095 (±0.076)	0.270 (±0.079)	<0.001^*^
Knee extension^a^	0.226 (±0.081)	0.192 (±0.093)	0.331 (±0.086)	<0.001^*^
Ankle dorsiflexion^b^	0.143 (0–0.355)	0.093 (0–0.248)	0.202 (0–0.445)	<0.001^**^

a *Mean values (SD)*.

b *Median values (range)*.

* *Comparison across groups with one-way ANOVA*.

** *Comparison across groups with Kruskal–Wallis test*.

### GMFCS Group A vs. Group B

The results showed consistently significantly lower values of force profile in all muscle groups of group B than group A, with the exception of knee extension where the difference was marginally significant (Table 3).

**Table 3:** Comparison of normalized to weight force data between patients with Gross Motor Function Classification System (GMFCS) I–II and patients with GMFCS III–IV.

	**GMFCS I–II (*n* = 121)**	**GMFCS III–IV (*n* = 39)**	***p***
Hip flexion^a^	0.223 (±0.081)	0.145 (±0.014)	<0.001^*^
Hip extension^a^	0.305 (±0.094)	0.246 (±0.111)	0.001^*^
Hip adduction^a^	0.227 (±0.091)	0.171 (±0.099)	0.001^*^
Hip abduction^a^	0.202 (±0.081)	0.105 (±0.083)	<0.001^*^
Knee flexion^a^	0.157 (±0.087)	0.080 (±0.084)	<0.001^*^
Knee extension^a^	0.222 (±0.083)	0.190 (±0.095)	0.041^*^
Ankle dorsiflexion^b^	0.133 (0–0.355)	0.091 (0–0.197)	0.001^**^

a *Mean values (SD)*.

b *Median values (range)*.

* *Comparison across groups with t test*.

** *Comparison across groups with Mann–Whitney U test*.

### Muscle Force Deficit Analysis

**Figure 5:**
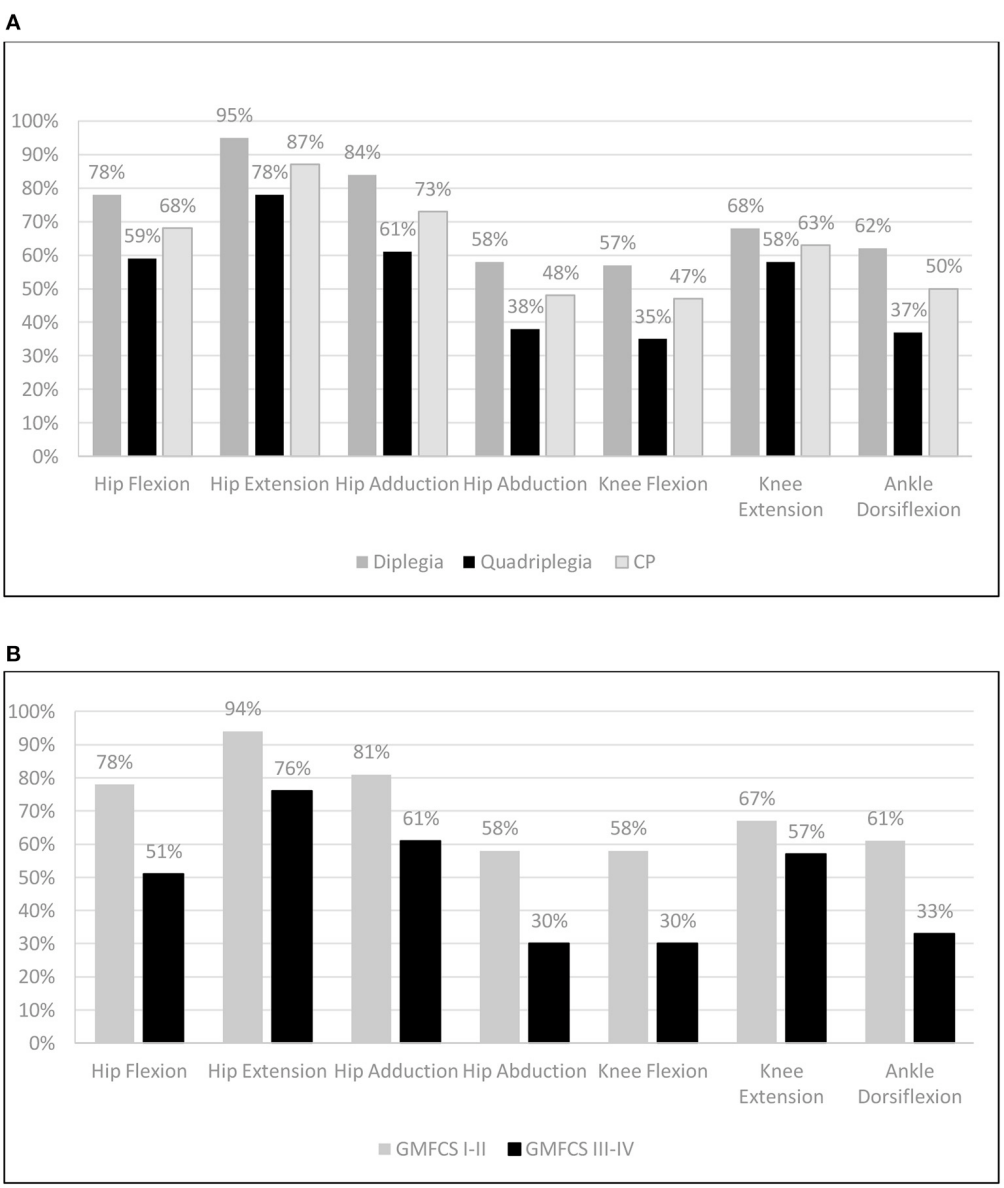
Mean muscle group force as percentage (%) compared to Typically Developing children: **(A)** per CP group and **(B)** per GMFCS group (Independent & Dependent) walkers.

Figure 5A demonstrates the deficit of the CP patients compared with the TD controls and Figure 5B shows the respective GMFCS groups A and B data. Our data revealed significant deficiencies in hip abductors (52%), in knee flexion (53%), and in ankle dorsiflexion (50%) for CP patients and lower values for knee extension (47%), hip flexion (32%), hip adduction (73%), and hip extension (13). For GMFCS group A, values ranged from 94 to 58% of the TD values, and for group B, values ranged lower in all muscle groups from 76 to 30%. The analysis of the antagonist muscle group couples revealed a large deficit imbalance between (adductors 27%) / (abductors 52%) and (knee extensors 37%) / (knee flexors 53%), and a smaller imbalance for the (hip extensors 13%) / (hip flexors 32%) couple. This pattern was evident in all age groups (Figure 4). Similarly, significant force deficiencies were identified in GMFCS III/IV patients as compared with TD children and GMFCS I/II patients.

## Discussion

In this cross-sectional study, we examined the age-dependent evolution of voluntary lower limb muscle strength in 160 patients with bilateral spastic CP, aged 7–16 years. When we compared their strength performance with typical developing children and adolescents of the same age, we were able to demonstrate that CP patients exhibited lower strength values in lower limb muscles when compared with their TD counterparts, with deficits being more pronounced in patients with quadriplegia and those with a more severe impairment (GMFCS levels III/IV vs. I/II). Furthermore, an important pattern of muscle strength imbalance between the antagonist muscles of the CP subjects was revealed.

Muscle weakness associated with the spastic form of CP has been studied extensively. Previous studies have linked insufficient force generation to decreased central activation or neuronal drive (5, 8, 20, 21), inappropriate co-activation of antagonist muscle groups (5, 8, 20), secondary myopathy (22–24), and altered muscle physiology (differing muscle force–frequency relationship and fatigue properties) (8), with considerable variation concerning the muscles involved and with a particular pattern of muscle weakness underlying the same type of gait (5, 6, 8, 9). Greater strength deficits in the lower extremities were recorded in distal muscles, when compared with the proximal ones (5, 6, 8, 20, 25) and in faster rather than slower speeds of movement (26). Similar motor deficits in distal muscles have been observed through adulthood, when they probably cause the compensatory trunkal–posture mechanisms (e.g., Trendelenburg sign) usually presented in adults with CP (27, 28).

The reliability for isometric force measurements of lower extremity muscles using handheld dynamometry in CP has been demonstrated (18, 29, 30). However, consensus is lacking with regard to standardization of the testing procedure (e.g., position of the child, method of assessment, with/without stabilization, peak/mean force, contraction time, intervals between measurement session, etc.) (19).

In our study, the slope of force development with age using absolute strength values moved in a positive direction (upwards), while it moved negatively (downwards) for normalized values, indicating that increases in BW through adolescence led to decreases in relative force in the tested muscles. Recently, in a prospective case series of 255 subjects (8–19 years old) with diplegic CP and in accordance with our results, it has been demonstrated, unlike absolute force values and weight that increase with age, that total lower extremity strength normalized to weight declines with increasing age (between 8 and 19 years), with a similar rate in young children and adolescents (10). As opposed to children with CP, TD participants in our study demonstrated a drop in the 13–14 age group in NF values, in all measured muscle groups. This coincides with a rather abrupt increase in BW; hence, this may be attributed to a possible increase of fat vs. muscle in their body composition (31) or to a relative lack of exercise as opposed to children with CP who were continuously enrolled in physiotherapy programs.

When comparing lower extremity strength in patients and TD controls of the same age, we demonstrated that in the majority of the muscles tested, CP patients exhibited significantly lower force measurements than their TD counterparts, and diplegic patients exhibited lower measurements than quadriplegics, with one exception: in hip extension, TD children and diplegic patients had similar values, higher though than quadriplegics. Reduced strength in all lower limb muscles tested has been reported previously (5, 32, 33). In accordance with our results, Thompson et al. also found that hip extensors preserved their strength in CP patients when compared with controls (9). They attributed this difference from other studies on the supine lying position that they chose (similar to our testing position), which is considered gravity neutral.

The force deficit was calculated for the whole group of CP patients. The average force deficit was 13–53%, with quadriplegics exhibiting larger deficits than diplegics (5–50% for the diplegics and 22–65% for the quadriplegics). At the muscle level, hip flexors and extensors, hip adductors, and knee extensors maintained forces nearer to TD controls (>60% TD), while hip abductors, knee flexors, and ankle dorsiflexors exhibited greater deficits (<50% TD). These results are in close approximation to the result of Dallmeijer et al. (32). In that study, mean strength values of children with CP and in the different GMFCS levels were compared with those of TD children, and it was reported that force measurements in knee extensors were reduced to 56–68% of values in TD children; in knee flexors, these were reduced to 36–68%; in hip abductors, to 47–76%; in hip flexors, to 63–82%; and in ankle plantar flexors, to 37–57% (32). Thompson et al. also reported similar deficits (43–90%), depending on the muscle group tested (9). In fact, our results were very similar to these authors for hip extensors and abductors, fairly close for the flexors and knee extensors, and different for the knee flexors, possibly due to a different measuring method. The knee extensors measured at 30° of flexion were relatively the weakest muscle group (9). Similarly, Stackhouse et al. found that children with spastic diplegic CP produced 56 and 73% less knee extensor and ankle plantar–flexor force, respectively, compared with participants without disabilities (8).

In our study, we also emphasized the distinct patterns of force development across age groups, for each pair of antagonist muscle groups. The imbalance in the NF values between the hip adductors and abductors was due to the fact that the former exhibited force close to TD values while the latter exhibited significantly lower values across all ages (Figure 4). A similar imbalance was noted between hip extensors and flexors where the former had values not significantly different from values in TD subjects while the latter were significantly lower in CP than in TD subjects. At the knee, both extensors and flexors had significantly reduced group forces compared with TD subjects; yet, the knee extensors tended to maintain force while the deficit for knee flexors was greater. These findings are relevant to the fact that our patients were able to stand and ambulate since one of the main mechanisms of maintaining erect posture during gait is the ability to extend the hip and knee (34). Remarkably, these imbalances were present in all age groups, indicating that the pattern of muscle involvement was constant independent of age and subject since this was a cross-sectional study and not a longitudinal one. These findings suggest that muscle imbalances are inherent to CP from an early stage and do not develop with age.

Important deficits were recorded in GMFCS III–IV patients when compared with controls (25%–70%), while GMFCS I–II patients performed better (58–94%). When we compared the strength in GMFCS levels I/II and III/IV to TD children, a uniform pattern emerged, with all patients exhibiting the best performance in hip extensors, closely followed by hip adductors, and the worst in knee flexors, hip abduction, and ankle dorsiflexion. We also demonstrated consistently lower values of force profile in all muscle groups of patients at GMFCS levels III/IV when compared with GMFCS levels I/II. There was a significant deficiency (<55% TD) in hip abductors, knee flexors, and ankle dorsiflexors for GMFCS levels III/IV compared with GMFCS levels I/II. Most previous studies investigating the relationship of lower limb strength and motor impairment (gross motor function) have demonstrated similar results, indicating that muscle strength affects walking ability (7, 9, 32). Thompson et al. depicted that with worsening ambulatory level from GMFCS levels I–III, strength values decreased in all muscle groups, while joint contractures increased. The greatest strength reduction between independent (GMFCS level I) and dependent walkers (GMFCS level III) was in the hip abductors (61%) and knee extensors at 30° (45%) (9). In their study, Dallmeijer et al. also reported that CP children with GMFCS level I had significantly higher strength values than GMFCS levels II and/or III for all muscle groups, except for knee extensors. The latter showed no differences between GMFCS levels (32). These results partly differ from our study, but our method of assessment in this muscle group and the calculation of normalized strength were different (9). Furthermore, the participation of patients with GMFCS level IV in our study may provide another explanation for that, since knee flexors have an important role in predicting mobility capacity or gait (32, 35), but those patients already had a severe walking impairment. In contrast to these studies presenting an association between lower limb force and walking performance, in a study of 24 children and adolescents (aged 5.3–19.6 years) with spastic CP, the authors failed to demonstrate similar force differences across GMFCS levels (36). However, in this study, researchers used a fixed handheld dynamometer and included patients with both unilateral and bilateral involvement, which may have affected their results.

The finding that, in children at GMFCS levels III/IV, the best performance was at the hip extensors, and that the strength of knee extensors was among the best preserved compared to controls, partly explain the fact that these patients were ambulatory up to the age of 16 years, as strength in hip and knee extensors better predicts their ability to walk, more so than spasticity (37). Recently, Davids et al. demonstrated that there is a 90% chance for independent ambulation (GMFCS levels I and II) when strength normalized for weight was 49% predicted relative to TD children, a 75% chance of independent ambulation when it was 33% predicted relative to TD children, and a 50% chance of independent ambulation when it was 16% predicted relative to TD children (10).

The major limitation of the our study was its cross-sectional design, which did not account for the effect of time and training on changes of weight, muscle mass, and force values. Secondly, our study group was fairly imbalanced: independent walkers were more represented, something that could influence the results. Another limitation of the study was the fact that while measuring CP patients' muscle forces, coactivation of the antagonist muscles occurred which may lead to underestimation of the strength of muscle measured (38). Finally, the accurate assessment of extremity muscle strength in children with CP was challenging due to a range of confounding variables, related both to patient's ability and motivation and to technical measurement issues, such as equipment used and testing protocol.

In conclusion, this study confirmed that NF in TD children was significantly greater than in children with CP in all muscle and age groups, with the greatest differences in the 15–16-year-old group. In CP, a constant pattern of significant muscle strength imbalance between antagonist muscles across all ages was identified, indicating that this muscle strength imbalance in CP was present from an early age and did not change significantly during development. Hip flexors, knee flexors, and ankle dorsiflexors were found significantly weaker than adductors and extensors. Lastly, a significant deficiency in hip abductors, knee flexors, hip flexors, and ankle dorsiflexors was identified in GMFCS III/IV when compared with TD and children in GMFCS levels I/II.

## Data Availability Statement

The raw data supporting the conclusions of this article will be made available by the authors, without undue reservation.

## Ethics Statement

Ethical review and approval was not required for the study on human participants in accordance with the local legislation and institutional requirements. Written informed consent to participate in this study was provided by the participants' legal guardian/next of kin.

## Author Contributions

ND: conceptualization, data collection, methodology-research design, resources, and assessment writing—original draft preparation. EN: formal analysis, research design, and writing—original draft preparation: MT: data collection, methodology, resources, and assessment. GG: resources and assessment. AP: conceptualization, methodology, writing—reviewing and editing, supervision. DP: data collection, methodology-research design, resources, and assessment. All authors contributed to the article and approved the submitted version.

## Conflict of Interest

The authors declare that the research was conducted in the absence of any commercial or financial relationships that could be construed as a potential conflict of interest.
